# Crop specific plant growth promoting effects of ACCd enzyme and siderophore producing and cynogenic fluorescent *Pseudomonas*

**DOI:** 10.1007/s13205-017-0602-3

**Published:** 2017-04-11

**Authors:** Priyanka, Toshy Agrawal, Anil S. Kotasthane, Ashok Kosharia, Renu Kushwah, Najam Waris Zaidi, U. S. Singh

**Affiliations:** 1 Department of Plant Pathology, Indira Gandhi Krishi Vishwavidyalaya, Krishak Nagar, Raipur, CG 492006 India; 2 Department of Plant Molecular Biology and Biotechnology, Indira Gandhi Krishi Vishwavidyalaya, Krishak Nagar, Raipur, CG 492006 India; 3 IRRI India Office, New Delhi, India

**Keywords:** ACCd enzyme, Confrontation assay, Fluorescent *Pseudomonas*, HCN, PGPR, Siderophore

## Abstract

Fluorescent *Pseudomonas*, aerobic, Gram-negative bacteria possess many traits that make them well suited as biocontrol and growth promoting agents. Our study revealed that isolates vary in mechanisms involved in the antagonist interactions against pathogen and growth stimulatory effects on host plant. Most of the potential antagonistic fluorescent *Pseudomonas* identified were avid iron chelators (P233, P201, 176, P76 and, P76). Wide variation in ACCd enzyme production was observed. ACCd enzyme assay tested P141 > P247 > P126, as potential ACCd enzyme producer. Cynogenic fluorescent *Pseudomonas* isolates P76 and P124 exerted strong inhibitory against *S. rolfsii.* However, another cynogenic fluorescent *Pseudomonas* P179 had no influence against *R solani* and *S. rolfsii* which remains unexplained. Noticeable crop specific plant growth stimulation exerted by different fluorescent *Pseudomonas* was observed on wheat (P124), chickpea (P72), lathyrus (P85, P216), greengram (P11), blackgram (P99, P233); bottlegourd (P248, P167); rice (P176, P247).

## Introduction

Aerobic gram negative Fluorescent *Pseudomonas* spp. have emerged as the largest and potentially most promising group of plant growth promoting rhizobacteria involved in the bio-control of plant diseases (Weller et al. 2002; Fravel 2005). A large number of secondary metabolites, growth hormones, antibiotics and chelating compounds such as siderophores (Choudhary et al. 2009; Beneduzi et al. 2012) are known to be released by these fluorescent pseudomonads. They maintain soil health by employing a wide variety of mechanisms including nitrogen fixation, enhanced solubilization of phosphate and phytohormone production (such as auxin and cytokinin). Plant growth-promoting rhizobacteria (PGPR) competitively colonize plant roots and stimulate plant growth and/or reduce the incidence of plant disease. Fluorescent *Pseudomonas* applied to seed or soil provides excellent control against plant pathogens (De La Fuente et al. 2006; Lagzian et al. 2013). *Pseudomonas* spp. produce an arsenal of antimicrobials (including hydrogen cyanide, HCN), pyoluteorin, phenazines, pyrrolnitrin, siderophores, cyclic lipopeptides and 2,4-diacetylphloroglucinol (DAPG) (Thomashow and Weller 1991; Weller 2007). This is considered as an indirect strategy to promote plant growth as well as the ability to induce systemic resistance in plants (Santoyo et al. 2012; Glick 2014). In this study, we evaluated the fluorescent isolates for siderophore and HCN production. PGPR and their interactions with plants are exploited commercially (Podile and Kishore 2007) and hold great promise for sustainable agriculture. Applications of these associations were investigated in Wheat (*Triticum aestivum*), Chickpea (*Cicer arientinum*), Lathyrus (*Lathyrus sativus*), Greengram (*Vigna radiata*), Blackgram (*Vigna mungo*), Bottlegourd (*Lagenaria siceraria*) and Rice (*Oryza sativa*) through seed bacterization.

## Materials and methods

### Microorganisms and culture conditions

The experimental material consisted of purified twenty-four isolates (Table 1) of fluorescent *Pseudomonas* spp. from soils (rhizospheric and non-rhizospheric) of different geographical locations of Chhattisgarh. Isolation of fluorescent pseudomonads was done by adopting serial dilution method on King’s B (KB) medium. Isolates were characterized on the basis of biochemical tests as per the procedures outlined in Bergey’s Manual of Systematic Bacteriology (Sneath 1986). Glycerol stock of isolates were maintained in the culture collections of the Department of Plant Molecular Biology and Biotechnology, Indira Gandhi Krishi Vishwavidyalaya, Raipur, Chhattisgarh, India and revived on KMB slants when required. Fungal pathogens *Rhizoctonia solani* and *Sclerotium rolfsii* were isolated from naturally infected sick soils of rice and chickpea and maintained on PDA slants.

**Table 1:** Fluorescent *Pseudomonas spp.* isolates used in the present study

S. no.	Isolates		Origin/location
1	P5	Fluoresecent *Pseudomonas*	Fenugreek soil, IGKV Horticulture, Raipur
2	P6	*Pseudomonas putida*	Cashew tree soil, IGKV Horticulture, Raipur
3	P11	*Pseudomonas putida*	Brinjal plant soil, IGKV Horticulture, Raipur
4	P67	*Pseudomonas fluorescens*	Charama
5	P72	*Pseudomonas putida*	Chhati
6	P76	*Pseudomonas putida*	Mustard field soil, chhati
7	P85	*Pseudomonas aeruginosa*	Cow dung soil, Darba
8	P99	Fluoresecent *Pseudomonas*	Jagtara
9	P124	Fluoresecent *Pseudomonas*	Kanker forest-3
10	P126	*Pseudomonas putida*	Kanker forest soil
11	P129	*Pseudomonas putida*	Kanker forest soil
12	P141	*Pseudomonas putida*	Kirda
13	P143	*Pseudomonas putida*	Kodebor
14	P151	*Pseudomonas putida*	Kurud
15	P161	*Pseudomonas putida*	VIP nursery soil, Raipur
16	P167	*Pseudomonas putida*	Purur
17	P176	*Pseudomonas putida*	Rice field, fallow land soil, Raipur
18	P179	Fluoresecent *Pseudomonas*	Rice-lathyrus field soil, Raipur
19	P201	*Pseudomonas putida*	VIP road, Raipur
20	P205	*Pseudomonas putida*	Babool tree soil, VIP road, Raipur
21	P216	Fluoresecent *Pseudomonas*	Bamboo tree soil, VIP road, Raipur
22	P233	Fluoresecent *Pseudomonas*	Badi, Tekabheta
23	P247	Fluoresecent *Pseudomonas*	Laichopi, Raweli
24	P248	*Pseudomonas aeruginosa*	Maize, Parsada

### Siderophore production

Qualitative and quantitative estimation of siderophore production was done by CAS assay (Schwyn and Neilands 1987). Specific tests were carried out for the identification of hydroxamate and catecholate types of siderophores following the standard methods (Arnow 1937). For qualitative estimation, chrome azurol S solution was prepared and added to melted King’s B agar medium in the ratio 1:15. Spot inoculation at the centre of the CAS plate was done from actively growing cultures of *Pseudomonas*. Colonies exhibiting an orange halo after 3 days incubation (28 ± 2 °C) were considered positive for siderophore production and the diameter of the orange halo was measured. Simultaneously succinate medium (broth) was also used for qualitative estimation of siderophore production on the basis of fluorescence observed after 3 days incubation (28 ± 2 °C).

#### Quantitative spectrophotometric assay for siderophore production (liquid assay)

For siderophore quantification, actively growing cultures of *Pseudomonas* was inoculated to 20 mL King’s B broth in 100 mL flasks and incubated for 3 days at 28 ± 2 °C. The bacterial cells were removed by centrifugation at 3000 rpm for 5 min. 0.5 mL of the culture supernatant was then mixed with 0.5 mL of CAS solution and 10 µl shuttling reagent (sulfosalicyclic acid). After 20 min of incubation, the absorbance of colour obtained was determined using spectrophotometer at 630 nm. Un-inoculated King’s B broth was used as blank while reference solution was prepared by adding CAS dye and shuttle solution to King’s B and absorbance was recorded. Values of siderophore released in King’s B was expressed as percent siderophore units and calculated using the formula: (*A*_r_ − *A*_s_)/*A*_r_ × 100; where *A*_r_ is the absorbance of reference solution and *A*_s_ is the absorbance of samples.

#### Hydroxyquinoline mediated siderophore test

Isolates were inoculated on King’s B medium supplemented with a strong iron chelater 8- Hydroxyquinoline (50 mg/L) (De Brito et al. 1995) and incubated at 28 ± 2 °C for 48–72 h. Only those bacteria that produce a more avid iron chelator will grow.

#### Arnow’s assay

Arnow’s assay was used for qualitative determination of catechol type of siderophore. Actively growing cultures of *Pseudomonas* were inoculated to 20 mL King’s B broth in 50 mL tubes and incubated for 3 days at 28 ± 2 °C. The bacterial cells were removed by centrifugation at 3000 rpm for 5 min. Three milliliter of the culture supernatant was then mixed with 0.3 mL of 5 N HCl solution, 1.5 mL of Arnow’s reagent (10 g NaNO_2_, 10 g Na_2_MoO_4_·2H_2_O dissolved in 50 mL distilled water) and 0.3 mL of 10 N NaOH. After 10 min the presence or absence of pink colour was observed and noted.

#### Tetrazolium test

This test is based on the capacity of hydroxamic acid to reduce tetrazolium salt by hydrolysis of hydroxymate groups using a strong alkali. The reduction and release of alkali shows red colour to a pinch of tetrazolium salt when 1–2 drops of 2 N NaOH and 0.1 mL of test sample is added. Instant appearance of a deep red colour indicated the presence of hydroxamate siderophore.

#### FeCl_3_ test

One milliliter of the culture supernatant was mixed with freshly prepared 0.5 mL of 2% aqueous FeCl_3_ and observed for the presence and absence of deep red colour.

### Confrontation assay

Fluorescent *Pseudomonas* isolates were multiplied on King’s B broth and incubated for two days at 28 °C till the fluorescent pigment appeared in the broth. Petri-plates containing pre-sterilized potato dextrose agar (PDA) medium was inoculated with plant pathogenic fungi *Sclerotium rolfsii* or *Rhizoctonia solani* (in the center) and incubated at 25 °C for three days till the fungus completely covered the entire plate. Bipartite interactions were performed following a simple confrontation assay technique as proposed by Kotasthane et al. (unpublished results), wherein edge of glass funnel was deployed for deposition of bio-agent surrounding pre-inoculated fungal pathogen.

### HCN production

The production of HCN was estimated by method of (Wei et al. 1991). The cultures were grown on KM plates supplemented with 4.4 g/L glycine as a precursor and the filter paper strips soaked in saturated picric acid solution were exposed to the growing *Pseudomonas* isolates. The plates were incubated for 7 days at 28 ± 2 °C and observations were recorded as change in the colour of filter paper to brown as positive indicator for HCN production.

### Quantitative estimation of ACC deaminase activity

ACC deaminase activity was determined by measuring the production of α-ketobutyrate and ammonia generated by the cleavage of ACC by ACC deaminase (Honma and Shimomura 1978; Penrose and Glick 2003). *Pseudomonas* isolates were grown in 5 mL of trypticase soya broth at 28 °C until they reached stationary phase. The cells were collected by centrifugation, washed twice with 0.1 M Tris–HCl (pH 7.5), re-suspended in 2 mL of modified DF minimal medium supplemented with 2 mM final concentration of ACC. Incubated at 28 °C with shaking for another 36–72 h. The induced bacterial cells were harvested by centrifugation at 3000*g* for 5 min, washed twice with 0.1 M Tris–HCl (pH 7.5), and re-suspended in 200 μL of 0.1 M Tris–HCl (pH 8.5). The cells were labilized by adding 5% toluene (v/v) and then vortexed at the highest speed for 30 s. Fifty microlitre of labilized cell suspension was incubated with 5 μL of 0.3 M ACC in an microcentrifuge tube at 28 ± 2 °C for 30 min. The negative control for this assay consisted of 50 μL of labilized cell suspension without ACC, while the blank consisted of 50 μL of 0.1 M Tris–HCl (pH 8.5) with 5 μL of 0.3 M ACC. The samples were then mixed thoroughly with 500 μL of 0.56 N HCl by vortexing and the cell debris was removed by centrifugation at 12,000*g* for 5 min. A 500 μL aliquot of the supernatant was transferred to a glass test tube, mixed with 400 μL of 0.56 N HCl and 150 μL of DNF solution (0.1 g 2,4-dinitrophenylhydrazine dissolved in 100 mL of 2 N HCl); and incubated at 28 °C for 30 min. One milliliter of 2 N NaOH was added to the sample before the absorbance at 540 nm was measured. The concentration of α-ketobutyrate in each sample was determined by comparison with a standard curve generated as follows: A stock solution of 100 mmol/L α-ketobutyrate (Sigma-Aldrich Co., Mumbai, India) was prepared in 0.1 mol/L Tris–HCl (pH 8.5) and stored at 4 °C. Just prior to use stock solution is diluted with same buffer to make a 10 mmol/L solution from which a standard concentration curve is generated. Each 500 μL α-ketobutyrate solutions of 1, 5, 10, 15, 20, 25, 30, 35, 40, 45 and 50 µM (prepared from stock solution) were mixed, respectively, with 400 μL of 0.56 N HCl and 150 μL DNF solution. One milliliter of 2 N NaOH was added and the absorbance at 540 nm was determined as described above. The values for absorbance versus α-ketobutyrate concentration (µM) were used to prepare a standard curve.

### Estimation of PGPR activity

The cultures of fluorescent *Pseudomonas* spp. were inoculated in 100 mL conical flask containing 25 mL King’s B broth and incubated at 28 ± 2 °C for 48 h. For field trials seed bacterization was done with stock cultures (used for confrontation assays) of selected fluorescent Pseudomonas isolate. Slurry for seed bacterization was prepared @ 5 mL of bacterial culture +3 g of talcum powder/kg of seed. Care was taken for uniform coating of all the seeds, which were dried in shade. Seeds of each of Wheat (GW-272), Chickpea, Lathyrus (KH-014), Greengram (puspa vishal), Blackgram (T-U-94-2), Bottlegourd (*Lagenaria siceraria*), Rice (swarna) were planted in pots containing soil mixed with sand and compost in the ratio of 3:1:1. Plants growth were measured for root and shoot length.

### Statistical analysis

The design of all phenotype assays and plant growth experiments was a completely randomized block with three replications per treatment. Data of all biochemical tests and plant growth experiments were subsequently analyzed by ANOVA followed by Duncan’s test using WASP (Web Agri Stat Package) software (http://icargoa.res.in/wasp/index.php). Critical difference at 0.05 level of significance was calculated for the observed values along with average and standard deviation. Duncan’s test controls the Type I comparison wise error rate and as per Duncan’s grouping, means with the same letter are not significantly different. Duncan’s test can be used irrespective of whether F is significant or not and compares all possible pairs of treatment means.

## Results

Isolates were characterized on the basis of biochemical tests as per the procedures outlined in Bergey’s Manual of Systematic Bacteriology (Sneath 1986) and tests reported by (Blazevic et al. 1973) that specifically differentiate the fluorescent *Pseudomonas* into *P. aeruginosa, P. putida* and *P. fluorescence* (Table 1).

### Qualitative and quantitative assay for siderophore production

**Fig. 1:**
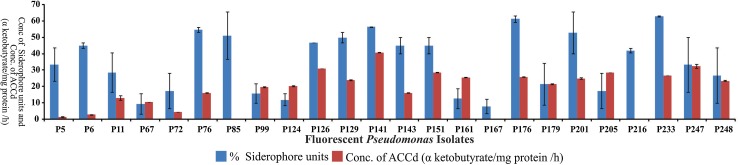
Percent Siderophore units and ACCd enzyme producing ability of different fluorescent *Pseudomonas*

Twenty-four isolates of fluorescent *Pseudomonas* were screened by different siderophore assay. All fluorescent *Pseudomonas* isolates produced siderophore on iron deficient succinate medium with variable chromogenic response and were also +ve for siderophore production on CAS agar plate assay and hydroxamate type of siderophore assay (tetrazolium and FeCl_3_ test). Siderophores are also viewed as contingent antibiotics, the selective advantage is easily verified by placing a bacterial culture in a medium containing a strong iron chelator 8-hydroxyquinoline (50 mg/L); only those bacteria that produce a more avid iron chelator grow. All fluorescent *Pseudomonas* isolates except P5, P11, P67, P72, P99, P126, P161, P167, P179 and P205 were +ve in HQ test. Fluorescent pseudomonas isolates P6, P85 and P233 produce a more avid iron chelator and produced high % siderophore units in quantitative test. Arnow’s assay (test for catechol type of siderophore) tested +ve for 22 isolates except P85 and P216. Fluorescent pseudomonas isolates P233 > P176 > P141 > P76 > P201 were all avid iron chelators and tested +ve for all siderophore tests (Fig. 1; Table 2).

**Table 2:** Siderophore, ACCd enzyme, HCN producing ability and inhibitory effect of fluorescent *Pseudomonas* isolates against *R solani* and *S. rolfsii*

	Treatment	Siderophore		ACC (α ketobutyrate/mg protein/h)	HCN	Confrontation assay (% inhibition)	
		% Siderophore units	HQ Test			*R. solani*	*S. rolfsii*
1	P5	33.54^bcdefg^ ± 10.21	–	1.48 ± 0.23	–	38.90^abc^ ± 11.10	19.45^ef^ ± 2.75
2	P6	45.1^abcde^ ± 1.77	***	2.9 ± 0.15	–	51.10^ab^ ± 1.10	35.55^abcde^ ± 6.65
3	P11	28.67^cdefgh^ ± 11.97	–	13.11 ± 1.29	–	34.45^abc^ ± 12.75	27.25^bcdef^ ± 9.45
4	P67	9.48^gh^ ± 6.15	–	10.51 ± 0.06	–	37.25^abc^ ± 9.45	27.80^bcdef^ ± 0.05
5	P72	17.40^fgh^ ± 10.74	–	4.42 ± 0.07	–	42.20^abc^ ± 3.30	26.10^cdef^ ± 6.60
6	P76	54.79^ab^ ± 1.46	*	16.24 ± 0.05	+++	49.45^ab^ ± 7.25	49.40^a^ ± 5.00
7	P85	51.15^abcd^ ± 14.48	***	nd	–	40.00^abc^ ± 6.70	43.35^abc^ ± 4.45
8	P99	15.93^gh^ ± 5.93	–	19.75 ± 0.21	–	29.45^bcd^ ± 7.25	33.85^abcde^ ± 8.35
9	P124	11.98^gh^ ± 3.65	*	20.37 ± 0.25	+++	43.85^abc^ ± 11.65	35.00^abcde^ ± 7.20
10	P126	46.77^abcde^ ± 0.10	–	31.05 ± 0.046	–	36.10^abc^ ± 8.30	17.21^ef^ ± 15.0
11	P129	49.9^abcd^ ± 3.23	*	23.94 ± 0.32	–	42.20^abc^ ± 2.20	8.33^f^ ± 2.78
12	P141	56.46^ab^ ± 0.21	*	40.87 ± 0.08	–	40.00^abc^ ± 6.70	23.30^cdef^ ± 7.80
13	P143	45^abcde^ ± 5.00	*	16.27 ± 0.14	–	42.75^abc^ ± 1.65	27.80^bcdef^ ± 11.10
14	P151	45^abcde^ ± 5.00	*	28.64 ± 0.26	–	50.00^ab^ ± 0.05	21.65^def^ ± 10.55
15	P161	12.71^gh^ ± 6.05	–	25.61 ± 0.05	–	22.20^cd^ ± 0.05	10.00^f^ ± 1.10
16	P167	7.92^h^ ± 4.49	–	nd	–	35.00^abc^ ± 5.00	41.65^abcd^ ± 2.75
17	P176	61.36^a^ ± 1.98	*	25.89 ± 0.21	–	52.75^ab^ ± 8.35	32.20^abcde^ ± 7.70
18	P179	21.56^efgh^ ± 12.81	–	21.61 ± 0.27	+++	4.44^d^ ± 2.22	10.01^f^ ± 7.79
19	P201	52.82^abc^ ± 12.82	*	25.02 ± 0.37	–	55.00^a^ ± 5.00	25.00^cdef^ ± 7.20
20	P205	17.39^fgh^ ± 10.73	–	28.56 ± 0.02	–	4.44^d^ ± 2.22	22.20^def^ ± 0.05
21	P216	41.98^abcdef^ ± 1.35	**	nd	–	44.45^abc^ ± 5.55	47.20^ab^ ± 8.30
22	P233	62.92^a^ ± 0.42	***	26.75 ± 0.05	–	45.55^abc^ ± 12.55	32.80^abcde^ ± 6.10
23	P247	33.34^bcdefg^ ± 16.67	**	32.61 ± 1.22	–	41.65^abc^ ± 8.35	22.80^def^ ± 5.00
24	P248	26.875^defgh^ ± 16.88	**	23.55 ± 0.272	–	36.65^abc^ ± 25.55	47.20^ab^ ± 2.80
	Max.	62.915^a^ ± 0.42				55.00^a^ ± 5.00	49.40^a^ ± 5.00
	Min.	9.48^gh^ ± 6.15				4.44^d^ ± 2.22	8.33^f^ ± 2.78
	CD (0.01%)	34.198				–	27.70
	CD (0.05%)	25.230				25.39	20.45
	CV	4.247				32.08	34.60
	Fcal					2.17	2.03

Values are average of three replications; values after ± represents standard error; CV, coefficient of variance; CD, critical difference; Values are significant at 1 and 5% levels; As per Duncan’s grouping means with the same letter are not significantly different; HQ hydroxyquinoline test; *** Luxuriant/high growth; ** medium growth; * low growth; –, no growth; nd, not determined

### Screening of 1-aminocyclopropane-1-carboxylic acid deaminase (ACC Deaminase) containing fluorescent *Pseudomonas* isolates

Test for ACC deaminase activity revealed wide variation in quantified amount of α-ketobutyrate produced by different fluorescent *Pseudomonas* isolates (Fig. 1; Table 2) which allowed us to classify them as high, medium and low ACC deaminase enzyme producing groups. Group of fluorescent *Pseudomonas* isolates produced µmol α ketobutyrate/mg protein/h in the range of 40.87 ± 0.08 to 25.02 ± 0.37 and 23.94 ± 0.32 to 10.51 ± 0.06 were placed in high and medium ACC deaminase producing groups. Isolate P141 was the highest enzyme producer followed by P247 and P126 (Fig. 1; Table 2). Three isolates P216, P5, P72 were identified as low ACC deaminase producers.

### Hydrogen cyanide production by isolates of fluorescent *Pseudomonas* spp.

Out of 24 Pseudomonas isolates only three isolates P67, P124 and P179 turned the strip brown confirming +ve for HCN production.

### In vitro antagonistic activity of fluorescent ***Pseudomonas*isolates against*R. solani*and*S. rolfsii***

**Fig. 2:**
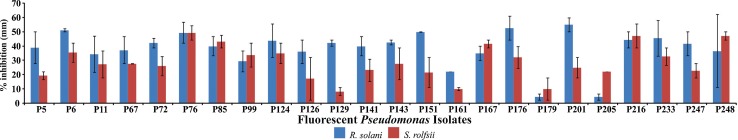
Inhibitory effect of fluorescent *Pseudomonas* isolates against *R solani* and *S. rolfsii*

Confrontation assays were performed to assess antagonistic potential of 24 fluorescent *Pseudomonas* isolates in vitro against *R. solani* and *S. rolfsii* (Fig. 2; Table 2). Differences in growth inhibitions of *R. solani* and *S. rolfsii* ranged from 4.44 to 55 and 8.325 to 49.4%, respectively. Confrontation assays revealed isolate P76 exerting antagonism against both *R solani* and *S rolfsii*, where as fluorescent *Pseudomonas* isolates (P201, 176, P6, P151 and P248, P216, P85, P167) exerted pathogen specific antagonism against *R. solani* and *S. rolfsii,* respectively. Isolate P205 and P129 showed the lowest inhibitory effect on *R. solani* and *S. rolfsii,* respectively. Variation in quantitative inhibitory data was significant at 0.01 and 0.05% level for S*. rolfsii* and 0.05% for *R. solani*.

### Correlation between siderophore production and in vitro antagonistic activity of fluorescent *Pseudomonas* isolates against *R. solani and S. rolfsii*

Some correlation between inhibitory effects and the ability to produce siderophore (quantitative assay) was observed. All potential fluorescent pseudomonas isolates identified following confrontation assays (P201, 176, P6, P151, P76 and P248, P216, P85, P167, P76 effective against *R. solani* and *S. rolfsii,* respectively) were high siderophore producers except P167 and P248. Fluorescent pseudomonas isolates P6, P85 and P233 produce a more avid iron chelator and produced high % siderophore units in quantitative tests of which P6 and P233 exhibited antagonistic activity against *R solani*. Similarly some correlation was also observed between antagonism and ability to produce HCN by fluorescent Pseudomonas. Isolates P76, P124, 179 were cynogenic of which P76 and P124 exerted strong inhibitory effects during confrontation assays against *S. rolfsii*, whereas P179 expressed no influence against these two soil borne fungal pathogens which remains unexplained.

**Fig. 3:**
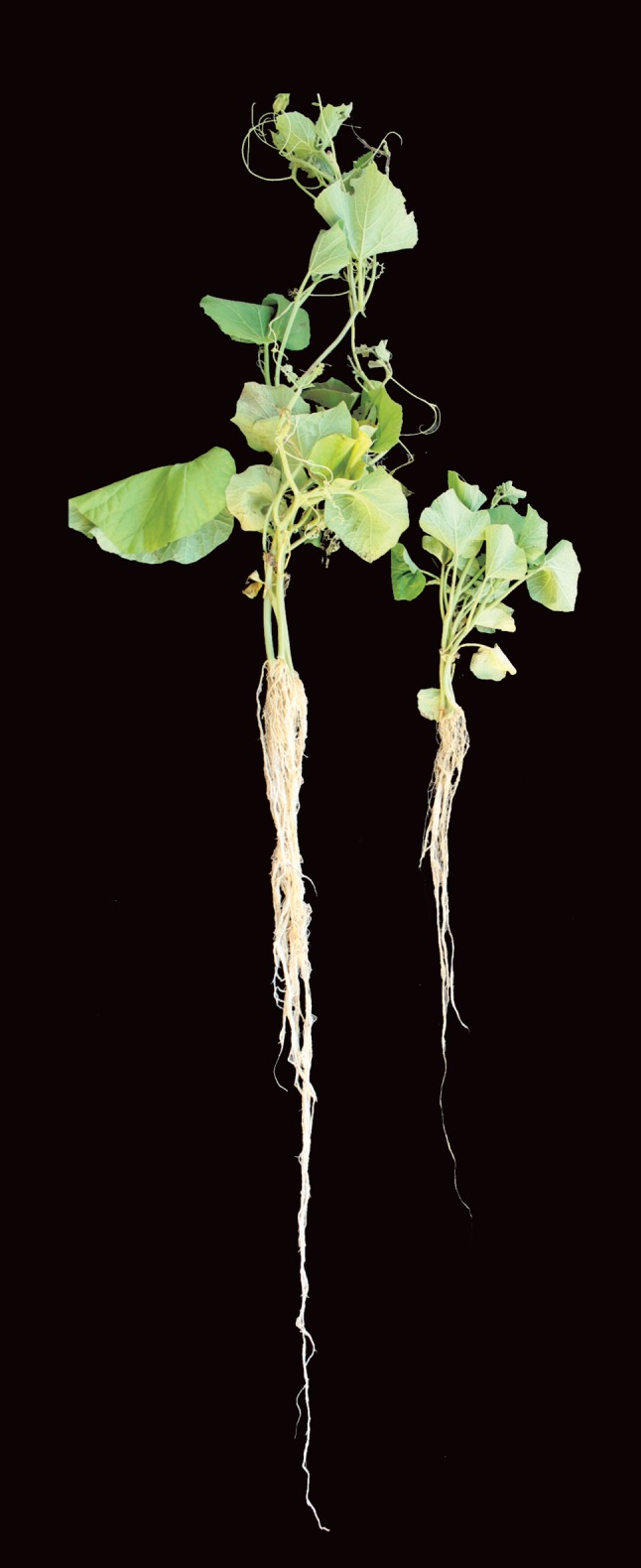
Plant growth promoting expression of bottle gourd seedling following seed bacterization with fluorescent *Pseudomonas* isolate

**Fig. 4:**
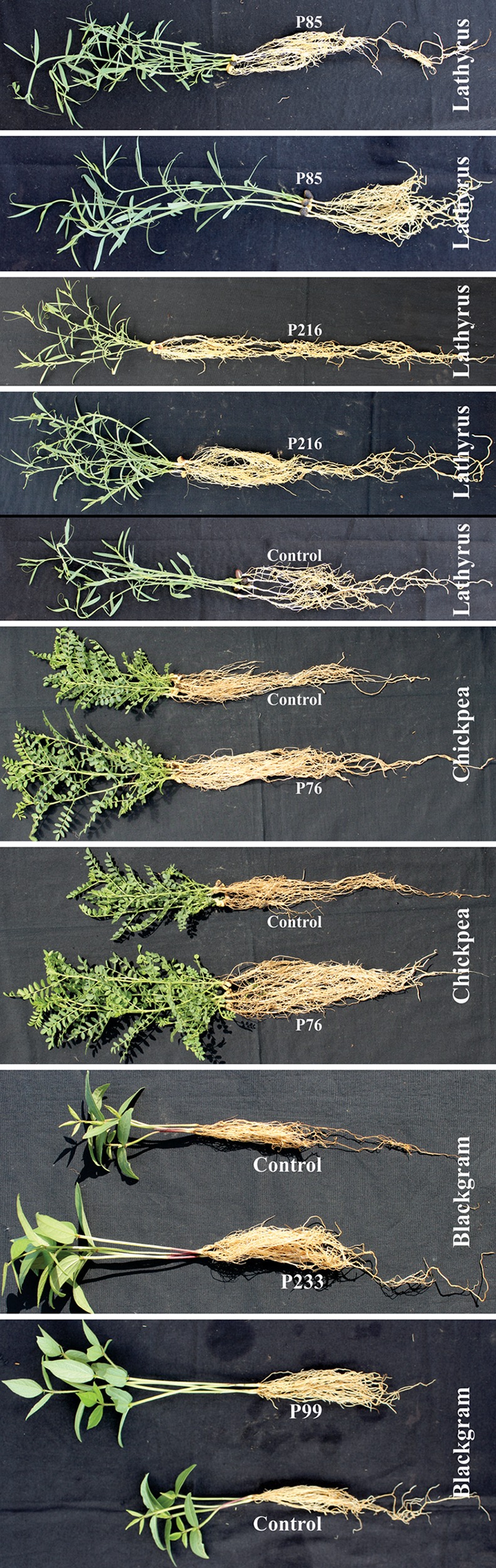
Plant growth promoting expression of bottle gourd seedling following seed bacterization with fluorescent *Pseudomonas* isolate black gram, chick pea and lathyrus seedling following seed bacterization with fluorescent *Pseudomonas* isolate

### Plant growth promoting response of rice, wheat, greengram, blackgram, lathyrus, chickpea, bottlegourd following seed bacterization with fluorescent *Pseudomonas* isolates

From pot experiments, plant growth-attributing characters such as root and shoot lengths were recorded for seven crops (rice, wheat, bottlegourd, lathyrus, chickpea, greengram, blackgram). Significantly greater amount of root and coleoptile growth stimulation was recorded in the seedlings of seven different crops derived following seed bacterization with 24 different fluorescent *Pseudomonas* isolates as compared to untreated control (Figs. 3, 4; Tables 3, 4). Effects amongst fluorescent *Pseudomonas* treatments to stimulate plant growth also varied. Plants of seven crop derived from bacterized seed had more stimulatory effects on coleoptile elongation than root length. Fluorescent Pseudomonas isolates P176 stimulated coleoptiles elongation on all seven crops tested. Stimulation of coleoptile elongation by fluorescent *Pseudomonas* isolates was more predominant in wheat followed by chickpea, lathyrus, greengram, blackgram, bottlegourd and rice.

**Table 3:** Plant growth promoting response of rice, wheat and bottle gourd seedlings following seed bacterization with fluorescent *Pseudomonas* isolates

S. no.	Treatment	Rice		Wheat		Bottlegourd	
		Root length (cm)	Shoot length (cm)	Root length (cm)	Shoot length (cm)	Root length (cm)	Shoot length (cm)
1	Control	9.325^efg^ ± 1.135	20.3^hijk^ ± 1.498	30.2^bcdefg^ ± 0.742	31.73^jk^ ± 0.4107	43.9^efg^ ± 12.27	17.6^efgh^ ± 1.254
2	P5	12.2^abcdef^ ± 1.301	20.775^ghijk^ ± 0.771	28.42^defgh^ ± 1.369	33.04^ij^ ± 0.6838	45.7^defg^ ± 6.74	14.33^fgh^ ± 0.794
3	P6	10.25^cdefg^ ± 0.487	21.65^fghij^ ± 1.713	27.68^efgh^ ± 1.685	30.77^k^ ± 1.0157	42.83^efgh^ ± 0.82	16.4^efgh^ ± 0.589
4	P11	8.5^fg^ ± 0.951	21.225^fghijk^ ± 0.684	31.86^bcdef^ ± 1.999	33.78^hi^ ± 0.6555	44.5^efg^ ± 6.01	11.1^fgh^ ± 0.733
5	P67	14.5^ab^ ± 2.480	23.225^abcdefg^ ± 0.390	27.24^fgh^ ± 1.259	36.04^efg^ ± 0.5142	54^cdefg^ ± 8.01	12.75^fgh^ ± 0.777
6	P72	10^defg^ ± 0.970	19.25^jk^ ± 0.811	27.56^fgh^ ± 1.755	36.35^defg^ ± 0.9332	83.3^b^ ± 1.15	15^fgh^ ± 1.780
7	P76	8.525^fg^ ± 0.581	21.85^efghi^ ± 0.835	30.66^bcdefg^ ± 0.897	36.75^cdef^ ± 0.8353	70.3^bc^ ± 2.77	36.73^bcd^ ± 5.981
8	P85	12.175^abcdef^ ± 1.035	24.45^abcd^ ± 0.362	27.19^gh^ ± 1.119	37.25^bcde^ ± 0.8825	41.83^fgh^ ± 12.44	40.68^bc^ ± 7.366
9	P99	9.2^efg^ ± 0.426	23.6^abcdef^ ± 0.942	27.69^efgh^ ± 1.102	38.84^ab^ ± 0.4621	70.8^bc^ ± 2.64	56.73^a^ ± 5.496
10	P124	10.3^cdefg^ ± 2.358	22.775^abcdefgh^ ± 0.536	36.52^a^ ± 2.766	38.18^abcd^ ± 1.1289	87.7^b^ ± 7.23	58.93^a^ ± 9.433
11	P129	10.825^bcdefg^ ± 0.409	20.135^ijk^ ± 0.613	28.12^efgh^ ± 1.907	38.55^abc^ ± 0.5677	69.88^bc^ ± 9.66	24.53^def^ ± 4.514
12	P126	9.775^efg^ ± 0.559	22.35^bcdefghi^ ± 0.598	30.07^bcdefg^ ± 1.316	36.89^bcdef^ ± 0.8538	64.95^bcdef^ ± 8.54	50.65^ab^ ± 12.99
13	P141	7.15^g^ ± 0.891	22.15^cdefghi^ ± 0.366	26.56^gh^ ± 1.240	39.78^a^ ± 0.9069	67.83^bcd^ ± 11.11	11.4^fgh^ ± 1.023
14	P143	7.625^g^ ± 1.004	19.025^k^ ± 1.424	31.14^bcdefg^ ± 1.775	35.9^efg^ ± 0.2994	59.18^cdefg^ ± 4.64	37.4^bcd^ ± 3.447
15	P151	13.625^abcd^ ± 2.755	24.725^ab^ ± 0.201	30.86^bcdefg^ ± 1.150	35.58^efgh^ ± 0.6486	75.63^bc^ ± 6.82	20.85^efg^ ± 0.296
16	P161	12.5^abcde^ ± 0.631	23.325^abcdefg^ ± 0.239	32.28^abcde^ ± 1.513	35.83^efg^ ± 0.4839	42.4^efgh^ ± 9.24	12.58^fgh^ ± 0.862
17	P167	9.8^defg^ ± 0.715	23.15^abcdefg^ ± 0.744	24.38^h^ ± 1.906	34.45^ghi^ ± 0.4381	73.5^bc^ ± 16.98	63.75^a^ ± 5.883
18	P176	10^defg^ ± 1.772	24.95^a^ ± 0.833	34.51^ab^ ± 1.602	38.27^abcd^ ± 0.6350	65.6^bcde^ ± 10.00	37.5^bcd^ ± 11.76
19	P179	7.8^g^ ± 0.356	22^defghi^ ± 0.082	28.61^defgh^ ± 1.749	36.09^efg^ ± 0.3703	20.33^h^ ± 1.13	3.95^h^ ± 0.466
20	P201	8.625^fg^ ± 2.138	21.4^fghijk^ ± 1.485	29.44^cdefg^ ± 1.610	38.59^abc^ ± 0.9865	52.88^cdefg^ ± 7.83	15.4^fgh^ ± 1.362
21	P205	7.55^g^ ± 0.144	22.325^bcdefghi^ ± 0.725	29.75^cdefg^ ± 1.447	36.81^cdef^ ± 0.3945	42.7^efgh^ ± 5.66	14.175^fgh^ ± 0.312
22	P216	8.7^efg^ ± 1.079	24.675^abc^ ± 1.071	27.41^fgh^ ± 1.529	35.16^fgh^ ± 0.2973	60^cdefg^ ± 9.37	11.55^fgh^ ± 0.689
23	P233	13.9^abc^ ± 2.200	24.325^abcde^ ± 0.531	32.82^abcd^ ± 1.641	35.86^efg^ ± 0.3638	41.58^gh^ ± 4.30	30.98^cde^ ± 12.056
24	P247	16^a^ ± 1.567	22.3^bcdefghi^ ± 1.219	33.79^abc^ ± 3.066	37.28^bcde^ ± 0.4847	44.5^efg^ ± 11.85	7.14^gh^ ± 0.865
25	P248	10^defg^ ± 0.799	20.825 ^ghijk^ ± 1.314	29.48^cdefg^ ± 1.389	38.12^abcd^ ± 0.5951	112.73^a^ ± 4.10	36.7^bcd^ ± 3.604
	Max.	16^a^ ± 1.567	24.95^a^ ± 0.833	36.52^a^ ± 2.766	39.78^a^ ± 0.9069	112.73^a^ ± 4.10	63.75^a^ ± 5.883
	Min.	7.15^g^ ± 0.891	19.025^k^ ± 1.424	24.38^h^ ± 1.906	30.77^k^ ± 1.0157	20.33^h^ ± 1.13	3.95^h^ ± 0.466
	CD 0.01	5.077	3.390	6.165	2.636	30.907	20.522
	CD 0.05	3.825	2.568	4.652	1.987	23.295	15.464
	C V	26.234	8.167	12.461	4.372	27.961	41.673
	F.cal**	2.987	3.412	2.814	9.65	5.559	10.136

Values are average of three replications; values after ± represents standard deviation; CV, coefficient of variance; CD, critical difference; ** Values are significant at 1 and 5% levels; As per Duncan’s grouping means with the same letter are not significantly different

**Table 4:** Plant growth promoting response of lathyrus, chickpea, green gram and black gram seedlings following seed bacterization with fluorescent *Pseudomonas* isolates

S. no.	Treat	Lathyrus		Chickpea		Greengram		Blackgram	
		Root length (cm)	Shoot length (cm)	Root length (cm)	Shoot length (cm)	Root length (cm)	Shoot length (cm)	Root length (cm)	Shoot length (cm)
1	Control	18.185^bcdefg^ ± 0.679	13.365^i^ ± 1.094	28.333^bcdef^ ± 0.726	16.333^mn^ ± 0.498	17.28^bcde^ ± 2.10	13.7^h^ ± 1.747	16.435^ef^ ± 0.733	12.625^mnop^ ± 0.3637
2	P5	20.565^abcde^ ± 0.957	16.635^cdefg^ ± 0.835	29^bcdef^ ± 6.449	14.933^n^ ± 0.536	15.39^de^ ± 2.58	14.665^fgh^ ± 0.830	18.565^bcdef^ ± 1.115	15.815^defgh^ ± 0.3044
3	P6	19.415^abcde^ ± 0.853	19.135^abc^ ± 0.382	20.533^fg^ ± 0.088	21.066^defghi^ ± 0.636	18.04^bcd^ ± 1.80	18.225^abcd^ ± 1.19	20.985^abcd^ ± 1.401	18.025^ab^ ± 0.5456
4	P11	22.575^ab^ ± 1.424	19.835^ab^ ± 0.521	29.833^bcde^ ± 0.441	22.866^bcde^ ± 0.754	18.03^bcd^ ± 0.44	20.385^a^ ± 0.987	19.925^bcde^ ± 1.858	16^defg^ ± 0.3969
5	P67	19.385^bcdef^ ± 2.496	13.885^hi^ ± 0.922	27.333^cdef^ ± 1.202	21.5^cdefg^ ± 1.510	14.72^de^ ± 0.48	17.625^bcde^ ± 0.468	20.215^bcde^ ± 1.540	13.375^jklmnop^ ± 0.742
6	P72	19.085^bcdef^ ± 1.464	16.2^defgh^ ± 0.450	38.85^a^ ± 6.438	25.7^a^ ± 0.115	17.47^bcde^ ± 1.12	16.1^defgh^ ± 1.022	20.225^bcde^ ± 2.244	13.165^lmnop^ ± 0.4625
7	P76	12.785 ^g^ ± 0.585	13.15^i^ ± 0.157	36.833^ab^ ± 2.920	21.266^defgh^ ± 0.504	13.99^e^ ± 2.29	14.8^fgh^ ± 0.452	19.15^bcde^ ± 1.187	14.575^hijk^ ± 0.3172
8	P85	18.285^bcdef^ ± 2.483	20.815^a^ ± 0.780	28.833^bcdef^ ± 0.833	23.233^bcd^ ± 0.536	16.22^cde^ ± 0.68	14.985^fgh^ ± 1.236	20.665^abcde^ ± 0.959	16.8^bcde^ ± 0.3007
9	P99	22.35^ab^ ± 3.126	17.765^bcdef^ ± 0.677	34.166^abc^ ± 2.351	21.7^cdefg^ ± 0.751	15.82^cde^ ± 1.29	19.125^abc^ ± 0.859	19.765^bcde^ ± 0.251	18.615^a^ ± 0.3912
10	P124	21.375^abcd^ ± 3.196	20.2^ab^ ± 0.774	26.833^cdef^ ± 4.086	23.833^abc^ ± 0.167	17.95^bcd^ ± 0.77	17.615^bcde^ ± 0.750	18.635^bcdef^ ± 0.884	15.235^fghi^ ± 0.6759
11	P126	16.375^defg^ ± 1.830	18.1^bcde^ ± 0.174	23^defg^ ± 2.021	20.75^efghi^ ± 1.010	15.99^cde^ ± 1.49	15.325^efgh^ ± 0.820	17.425^def^ ± 0.574	12.5^nop^ ± 0.3048
12	P129	16.465^defg^ ± 1.723	18.015^bcde^ ± 0.923	27.666^cdef^ ± 0.882	25.066^ab^ ± 0.924	15.69^cde^ ± 2.80	16.865^cdefg^ ± 1.170	19.715^bcde^ ± 1.178	14.025^ijklm^ ± 0.4498
13	P141	15.665^efg^ ± 2.070	14.8^ghi^ ± 1.093	33.166^abc^ ± 2.242	18.933^hijkl^ ± 0.470	22.15^a^ ± 0.76	16.4^defg^ ± 0.524	19.65^bcde^ ± 0.991	17.515^abc^ ± 0.4502
14	P143	17.55^bcdefg^ ± 0.484	15.285^fghi^ ± 0.739	33.65^abc^ ± 3.839	21^defghi^ ± 1.732	18.49^abcd^ ± 0.84	19.175^abc^ ± 1.301	22.135^abc^ ± 2.148	17.685^abc^ ± 0.4719
15	P151	13.965^fg^ ± 0.380	16.8^cdefg^ ± 1.365	31.433^abcd^ ± 2.599	21.066^defghi^ ± 0.581	19.59^abc^ ± 1.48	20.235^a^ ± 1.473	16.465^ef^ ± 0.802	12.335^op^ ± 0.4575
16	P161	15.775^efg^ ± 1.730	13.115^i^ ± 0.984	30.5^abcde^ ± 2.180	22.166^cdef^ ± 0.928	15.94^cde^ ± 0.38	16.185^defg^ ± 0.648	16.785^def^ ± 1.574	13.275^klmnop^ ± 0.2087
17	P167	18.735^bcdef^ ± 0.671	16.85^cdefg^ ± 1.646	22^efg^ ± 4.726	16.833^lmn^ ± 0.899	18.63^abcd^ ± 1.27	16.465^defg^ ± 0.390	17.835^def^ ± 1.218	13.885^ijklmn^ ± 0.3319
18	P176	20.525^abcde^ ± 2.771	18.785^abcd^ ± 1.573	24.25^defg^ ± 3.320	19.5^ghijk^ ± 1.155	17.08^bcde^ ± 0.23	19.675^ab^ ± 0.338	22.7^ab^ ± 2.257	17.165^bcd^ ± 0.6932
19	P179	16.7^cdefg^ ± 1.198	13.365^i^ ± 0.546	16.75^g^ ± 1.299	18.25^jklm^ ± 0.722	20.37^ab^ ± 0.17	16.065^defgh^ ± 0.228	17.75^def^ ± 2.027	16.5^cdef^ ± 0.3857
20	P201	19.915^bcde^ ± 3.689	16.065^efgh^ ± 0.933	24.4^defg^ ± 3.114	19.733^ghijk^ ± 0.536	16.47^bcde^ ± 1.08	16.935^cdef^ ± 0.388	19.885^bcde^ ± 1.499	16.75^bcde^ ± 0.4392
21	P205	18.615^bcdef^ ± 0.742	16.525^defg^ ± 0.545	23.933^defg^ ± 0.788	20.166f^ghij^ ± 0.601	16.60^bcde^ ± 1.80	14.45^gh^ ± 0.149	16.95^def^ ± 1.551	14.175^ijkl^ ± 0.3099
22	P216	25.7^a^ ± 2.957	15.185^fghi^ ± 0.508	29.866^bcde^ ± 4.332	20.766^efghi^ ± 0.865	18.00^bcd^ ± 1.41	16.965^cdef^ ± 0.889	19.675^bcde^ ± 2.523	14.7^ghij^ ± 0.8147
23	P233	15.715^efg^ ± 1.047	14.65^ghi^ ± 0.899	28.666^bcdef^ ± 0.441	22.5^cdef^ ± 1.155	17.84^bcde^ ± 1.07	18.065^abcd^ ± 0.268	24.825^a^ ± 2.510	15.585^efgh^ ± 0.8166
24	P247	19.315^bcdef^ ± 1.818	19.825^ab^ ± 1.567	31.4^abcd^ ± 2.358	18.733^ijkl^ ± 0.617	16.00^cde^ ± 0.97	16.65^defg^ ± 0.388	18.125^cdef^ ± 1.196	15.7^efgh^ ± 0.4449
25	P248	21.93^abc^ ± 2.049	20.705^a^ ± 0.755	26.033^cdef^ ± 2.338	17.366^klm^ ± 0.857	16.09^cde^ ± 1.12	15.2^efgh^ ± 0.228	14.585^f^ ± 0.716	16.8^bcde^ ± 0.6334
	Max.	25.7^a^ ± 2.957	20.815^a^ ± 0.780	38.85^a^ ± 6.438	25.7^a^ ± 0.115	22.15^a^ ± 0.76	20.385^a^ ± 0.987	24.825^a^ ± 2.510	18.615^a^ ± 0.3912
	Min.	12.785^g^ ± 0.585	13.115^i^ ± 0.984	16.75^g^ ± 1.299	14.933^n^ ± 0.536	13.99^e^ ± 2.29	14.665^fgh^ ± 0.830	14.585^f^ ± 0.716	12.335^op^ ± 0.4575
	CD 0.01	7.243	3.447	11.443	3.190	–	3.268	5.700	1.857
	CD 0.05	5.467	2.590	8.584	2.391	3.923	2.464	4.298	1.401
	C V	20.760	10.994	18.490	7.075	16.217	10.361	15.917	6.495
	Fcal**	2.274	7.27	2.835	9.781	1.688	4.488	2.115	14.069

Values are average of three replications; values after ± represents standard deviation; CV, coefficient of variance; CD, critical difference; ** Values are significant at 1 and 5% levels; as per Duncan’s grouping means with the same letter are not significantly different

#### Potential isolates stimulating plant growth (coleoptiles elongation and/or root length) in different crops were as follows

Wheat (GW-272): P124 stimulated coleoptiles elongation by 20.23% and root length by 17.30%; Chickpea (*Cicer arientinum*): P72 stimulated coleoptiles elongation 36.45% and root length by 27.08%; Lathyrus (KH-014): P85 stimulated coleoptiles elongation by 35.78% and P216 stimulated root length by 29.22%; Greengram (puspa vishal): P11 stimulated coleoptiles elongation by 32.81%; Blackgram (T-U-94-2): P99 stimulated coleoptiles elongation 32.16% and P233 stimulated root length by 33.8%; Bottlegourd (*Lagenaria siceraria*): P248 stimulated coleoptiles elongation 72.39% and P167 root length by 68.83%; Rice (swarna): P176 stimulated coleoptiles elongation 16.56% and P247 stimulated root length by 41.69%. Fluorescent *Pseudomonas* isolates stimulating both coleoptiles elongation and/or root length were P141 on greengram; P6, P143, P176 and P233 on blackgram; P76, P99, P124 and P167 on bottlegourd; and P151, P233 on rice. Fluorescent *Pseudomonas* isolates stimulating only root length were: P72, P129, P141 and P151 on bottlegourd; and P67 and P247 on rice.

## Discussion

Understanding the mechanisms involved in the antagonist interactions between bacteria, pathogen and host plant is important for efficient utilization of these natural resources in crop health management (Thomashow and Weller 1991). In soil, plant roots normally coexist with bacteria and fungi which may produce siderophores capable of sequestering the available soluble iron and hence interfere with plant growth and function. Siderophore production confers competitive advantages to PGPR that can colonize roots and exclude other microorganisms from this ecological niche (Haas and Défago 2005). Under highly competitive conditions, the ability to acquire iron via siderophores may determine the outcome of competition for different carbon sources that are available as a result of root exudation or rhizo deposition (Crowley 2006). Siderophores production by strains of *Pseudomonas* spp. for plant disease control is of great interest because of its possibilities in the substitution of chemical pesticides. In this study, we have compared the ability of several fluorescent *Pseudomonads* to produce suderophores, cyanogenesis and antagonism in plate assay. All potential fluorescent *Pseudomonas* isolates identified following confrontation assays were high siderophore producers except P167, P248. Of the potential antagonistic fluorescent *Pseudomonas* isolates P233, P201, P176, were effective against *R. solani* where as P76 against both *R solani* and *S. rolfsii* were avid iron chelators and high siderophore producers. Similarly, microbial cyanogenesis has been demonstrated in a few bacterial species (belonging to the genera *Pseudomonas, Chromobacterium, Rhizobium* and several cyanobacteria (Blumer and Haas 2000). Glycine has generally been used as a precursor of cyanide in fungi and bacteria (Brysk et al. 1969; Wissing 1974) and cyanogenesis is one of the mechanisms of antagonism and biocontrol properties (Haas and Défago 2005; Lanteigne et al. 2012). In this investigation identified cynogenic isolates P76 and P124 exerted strong inhibition against *S. rolfsii* where as cynogenic P179 was ineffective against *R solani* and *S. rolfsii* remains unexplained. Our study revealed that isolates vary in the mechanisms and ability to inhibit pathogens. Plant growth-promoting bacteria use a number of different mechanisms to promote the growth of plants (Glick 2012), but enzyme 1-aminocyclopropane-1-carboxylate (ACC) deaminase producing strains are the key bacterial trait which relives plants from deleterious effects of ethylene by cleaving ACC, into ammonia and –ketobutyrate (Honma and Shimomura 1978) and also facilitates plant growth (Glick et al. 2007; Glick 2014). ACC deaminase activity revealed wide variation in ACCd enzyme production in the range of 40.87 ± 0.08 to 25.02 ± 0.37 and 23.94 ± 0.32 to 10.51 ± 0.06 µmol α ketobutyrate/mg protein/h and P141 > P247 > P126, were potential ACCd enzyme producer. Fluorescent *pseudomonas* are one of the most abundant bacteria in the rhizosphere of many plants (Botelho and Mendonça-Hagler 2006), have large capacity to produce phytohormones, mainly auxins (Patten and Glick 2002a, b; Khalid et al. 2005) and secondary metabolites, such as antibiotics (Bergsma-Vlami et al. 2005), thus they are able to improve plant growth and plant health (Belimov et al. 2009a, b). Seed (of crops) biopriming with different isolates of fluorescent pseudomonas with ability to produce different levels of ACCd enzyme and siderophore were correlated with plant growth promoting effects. More stimulatory effects on coleoptile elongation than root length were observed. Our combined in vitro and pot experiment show the potential of isolate P176 to be developed as a commercial bio-inoculant as it stimulated coleoptiles elongation on all seven crops tested. Noticeable effects of plant growth stimulation were observed more on legume crops than on cereals. Potential isolates stimulating plant growth (coleoptiles elongation and or root length) specific to different crops were as follows: Both coleoptile elongation and root length:- Wheat (P124), Chickpea (P72); Only coleoptile elongation:- Greengram (P11), Lathyrus (P85), Blackgram (P99), Bottlegourd (P248), Rice (P176); Only root length:- Lathyrus (P216), Blackgram (P233), Bottlegourd (P167), Rice (P247). Nonetheless, this study and the results are particularly useful for identifying likely candidates for bio-control and for making educated guesses concerning the mechanisms by which they induce plant growth.
